# Duke activity status index is not predictive of outcomes after kidney transplantation: a retrospective observational study

**DOI:** 10.1186/s12882-025-04300-2

**Published:** 2025-07-04

**Authors:** Ruth Fergie, Alexander P. Maxwell, Aisling E. Courtney, Michael Corr, Stephen O’Neill, Emma L. Cunningham

**Affiliations:** 1 Centre for Public Health, Queen’s University Belfast, Belfast, UK; 2 Regional Nephrology & Transplant Unit, Belfast City Hospital, Belfast, BT9 7AB UK

**Keywords:** Kidney transplant, End-stage kidney disease, Functional capacity

## Abstract

**Background:**

Reduced functional capacity increases the risk of adverse outcomes after kidney transplantation. The Duke Activity Status Index is a measurement of physical function, previously reported as being predictive of adverse outcomes after major non-cardiac surgery. This study assessed the ability of the Duke Activity Status Index to predict adverse outcomes for patients undergoing kidney transplantation.

**Methods:**

Adult kidney transplant recipients with a Duke Activity Status Index calculated at time of listing for transplantation in Northern Ireland between 2019 and 2024 were analysed. Dichotomous outcomes (delayed graft function, unplanned critical care admission, 30-day hospital re-admission, 30-day severe postoperative complication, 30-day cardiovascular complication) were analysed using multivariate logistic regression. Post-transplant length of stay was assessed using multivariate linear regression. All-cause mortality and death-censored graft loss were evaluated using Cox proportional hazard regression models.

**Results:**

Data was available for 408 kidney transplant recipients. Duke Activity Status Index was not predictive of delayed graft function (aOR 0.99 (95% CI 0.66–1.01) *p* = 0.359), unplanned critical care admission (aOR1.00 (95% CI 0.97–1.04), *p* = 0.866), length-of-stay post-transplant, 30-day hospital re-admission (aOR1.01 (95% CI 0.99–1.03), *p* = 0.457), 30-day severe postoperative complication (aOR 1.01 (95% CI 0.99–1.03) *p* = 0.489), 30-day cardiovascular complication (aOR 0.99 (95% CI 0.93–1.06), *p* = 0.850), all-cause mortality (aHR 1.00 (0.96–1.04), *p* = 0.89) or death-censored graft loss (aHR 0.97 (95% CI 0.93–1.01), *p* = 0.14).

**Conclusions:**

In this cohort, the Duke Activity Status Index was not an independent predictor of short or long-term adverse outcomes following kidney transplantation. These findings suggest that the Duke Activity Status Index may have limited utility in assessing functional capacity in waitlisted kidney transplant candidates.

**Trial registration:**

Not applicable.

## Background

Recipients of kidney transplants with pre-existing reduced functional capacity have an increased risk of adverse outcomes after kidney transplantation. These include increased mortality, earlier graft loss, delayed graft function, prolonged length of stay, early hospital re-admission, immunosuppression intolerance and post-transplant delirium [1–10]. 

Determination of reduced functional capacity in patients who are being assessed for kidney transplantation aims to identify those at risk for adverse outcomes, counsel such patients on expected outcomes, aid decision-making on a candidate’s suitability for transplantation and consideration of which type of organ they may be suitable for, and identify patients who may benefit from prehabilitation [11, 12]. Traditionally, clinicians have relied on their subjective judgement of a patient’s functional capacity. However, evidence shows poor agreement between subjective assessments and validated measures [13, 14] and a limited ability of subjective assessments to predict adverse outcomes after major surgery [15].

There are many validated measures of functional capacity in the general population, including the six-minute walk test, short physical performance battery, cardiopulmonary exercise test, and Fried’s frailty phenotype score [16]. However, several factors have limited the implementation of these measures into clinical practice. Dedicated equipment, trained staff and a suitable amount of time are required. Additionally, there is a lack of consensus amongst transplant professionals regarding which tool is optimal for use and, when used, how to interpret the results of said measurement to inform management of the potential candidate being considered for waitlisting [17]. Furthermore, many of these measures have not been validated in patients with end-stage kidney disease (ESKD) [16].

The Duke Activity Status Index (DASI) is a self-administered 12-item questionnaire which was developed in a sample of 50 adults who underwent exercise testing in the USA. It was then validated in a further 50 adults. Results gave a score between 0 and 58.2, which was shown to correlate with peak oxygen uptake [18]. The scores were then approximated to a metabolic equivalent that the patient could achieve. DASI was further assessed in the landmark Measurement of Exercise Tolerance before Surgery (METS) study [19]. It compared methods of assessing cardiopulmonary fitness for participants undergoing major non-cardiac surgery. These included DASI, subjective assessment and cardiopulmonary exercise testing (CPET). Only DASI scores correlated with death or myocardial infarction 30 days post-operatively [19]. As a result of this, international guidelines now recommend using DASI to assess functional capacity in patients undergoing major non-cardiac surgery [20].

There has been increasing interest in using DASI scores in the process of waitlisting candidates for solid organ transplantation. It has recently been assessed in a cohort of candidates waitlisted for liver transplantation with DASI scores being predictive of all-cause mortality and waitlist mortality [21].

Using DASI in the waitlisting process for kidney transplantation could have many advantages including identifying patients at risk for adverse outcomes. It may also identify patients at lower risk for complications hence minimising unnecessary cardiovascular screening investigations and potentially reducing inequity of access to transplantation as a result of subjective judgements by the multidisciplinary team.

This study aimed to assess the ability of DASI to predict adverse outcomes after kidney transplantation for candidates who have been waitlisted.

## Materials and methods

### Study design

This was a retrospective cohort study using a prospectively designed clinical database – the Northern Ireland Kidney Transplant Database (ethics approval number: REC 23/NI/0034).

### Study participants

All patients aged 18 or over who received a kidney transplant in Northern Ireland from 2019 to 2024 and had a DASI score calculated at the time of listing for transplantation were included in the analysis. Recipients were followed up until death or September 13, 2024. Recipients who were non-residents in Northern Ireland were not included in the analysis due to the absence of follow-up data.

### Study procedures

All participants had completed the DASI questionnaire at the time of listing for transplantation. The DASI questionnaire consists of 12 items assessing a candidate’s ability to perform a variety of tasks (including activities of daily living and leisure activities) [18]. All responses are yes/no with a specific score given for each answer. Total scores range between 0 and 58.2 with higher scores indicating higher functional capacity.

### Data collection

Clinical data for all recipients and donors was prospectively collected by three clinicians working as part of the kidney transplant team.

#### Recipient variables

Recorded variables for recipients included age, sex, body mass index (BMI), smoking status, primary renal disease, mode of renal replacement therapy (RRT) at the time of transplantation, history of diabetes, history of cardiovascular disease, history of prior kidney transplantation, number of human leucocyte antigen (HLA) mismatches and cold-ischemic time. Primary renal disease is grouped into six categories: glomerulonephritis, tubulointerstitial disease, autosomal dominant polycystic kidney disease (ADPKD), diabetic nephropathy (DN), other specified aetiologies and chronic kidney disease (CKD) not defined. History of diabetes is defined as the presence of diabetes requiring oral agents or insulin therapy. History of cardiovascular disease requires objective evidence and physician documentation. For example, a history of ‘myocardial infarction’ would require physician documentation with a corresponding electrocardiogram, elevation in cardiac enzymes or record of subsequent confirmatory angiography. Included was coronary artery disease (myocardial infarction, symptoms requiring percutaneous intervention or bypass surgery), symptomatic valvular disease, arrhythmias, symptomatic left ventricular systolic dysfunction, symptomatic right ventricular systolic dysfunction, ischaemic stroke or peripheral vascular disease with intervention.

#### Donor variables

Recorded variables for donors include donor age, sex and type (living or deceased).

#### Outcomes

Outcomes recorded include length of stay post-transplant, delayed graft function, unplanned critical care admission, 30-day re-admission to hospital, 30-day severe postoperative complication, 30-day cardiovascular complication, all-cause mortality and death-censored graft loss.

Graft failure was defined as the commencement of an alternative mode of RRT. Delayed graft function was defined as a requirement for dialysis therapy in the first week after transplantation. 30-day severe postoperative complication was defined as the presence of grade 3–5 complications as classified by the Clavien-Dindo classification of surgical complications [22]. 30-day cardiovascular complications included myocardial infarction, atrial fibrillation with a fast ventricular response or decompensated heart failure.

### Statistical analysis

All analyses were conducted using SPSS version 29.0.2.0 (IBM Corp., Armonk, NY). Patient and donor characteristics were summarised using appropriate descriptive statistics.

Dichotomous outcomes (delayed graft function, unplanned critical admission, 30-day re-admission to hospital, 30-day severe post-operative complication, 30-day cardiovascular complication) were analysed using univariable logistic regression to calculate odds ratios (OR) and multivariable logistic regression to calculate adjusted odds ratios (aOR) and confidence intervals (CI). Linear outcomes (length of stay) were analysed using multivariable linear regression to calculate adjusted coefficients and CIs. Length of stay was log-transformed due to its skewed distribution. All-cause mortality and death-censored graft loss were evaluated using Cox proportional hazard regression models. To account for potential confounders, all analyses were adjusted for prognostic factors of postoperative mortality and morbidity after kidney transplantation. The following factors were selected a priori based on previous literature: age, sex, dialysis vs. pre-emptive transplantation, presence of diabetes, deceased donor vs. living donor transplantation and cold ischemic time. Further analysis of the METS study cohort has suggested that those with DASI scores more than 34 are at a reduced risk of adverse postoperative outcomes [19]. We therefore assessed the relationship between preoperative scores more than 34 against all outcomes of interest. Sub-group analyses were conducted to evaluate the predictive value of the DASI score for outcomes in both live donor recipients and deceased donor kidney transplant recipients. All statistical tests were two-sided with statistical significance set at < 0.05.

## Results

A total of 408 recipients met the inclusion criteria for participation in the study. Data was available for all recipients. All recipients were transplanted between 2019 and 2024.

### Demographics

The baseline characteristics for the 408 included recipients are described in Table 1. 191 (46.8%) recipients received kidneys from donors who were alive and 217 (53.2%) from donors who were deceased. The median DASI score at listing was 42.7 points (IQR 29.5–50.7). The median time from calculation of DASI score to transplantation was 9 months (IQR 4–16). Median follow-up time was 25 months post KT (IQR 13–38 months).

**Table 1:** Demographics of the 408 recipients in the study

Variable		
Age, median (IQR), years		54 (42–64)
Female, N (%)		145 (35.5)
BMI, median (IQR)		26 (23–30)
DASI score		42.7 (29.5–50.7)
		**N (%)**
Primary renal disease	Glomerular	107 (26.2)
	Interstitial	66 (16.2)
	ADPKD	51 (12.5)
	DN	61 (15)
	Other	76 (18.6)
	CKD not defined	47 (11.5)
RRT prior to transplant	Haemodialysis	180 (44.1)
	Peritoneal dialysis	61 (15)
	Pre-emptive	167 (40.9)
Co-morbidity	CV disease	82 (20.1)
	Diabetes	98 (24)
	Prior Transplant	82 (20.2)
Smoking status	Current	63 (15.4)
	Ex	95 (23.3)
	Never	246 (60.3)
	Missing	4 (1)
Transplant Details	HLA mismatches, median (IQR)	3 (2–4)
	Cold Ischaemic Time, median (IQR), minutes	482 (272–987)
Donor type	Deceased after brainstem death	112 (27.5)
	Deceased after cardiac death	105 (25.7)
	Living	191 (46.8)

Key: IQR = interquartile range. BMI = body mass index. DASI = Duke Activity Index Score. ADPKD = autosomal dominant polycystic kidney disease. CKD = chronic kidney disease. CV = cardiovascular. HLA = human leucocyte antigen

### Outcomes

Data regarding postoperative outcomes are described in Table 2. In univariate analysis, patients with a lower DASI score (that is worse pre-existing functional capacity) were significantly more likely to experience delayed graft function (OR 0.98 (95% CI 0.966–0.997), *p* = 0.019) and have a prolonged length of stay (Table 2). However, following adjustment for confounders, lower DASI scores were no longer predictive of delayed graft function (aOR 0.99 (95% CI 0.66–1.01), *p* = 0.359) or prolonged length of stay (Table 2). Of interest, further statistical analysis showed that patients with a lower DASI score were more likely to receive a transplant from a donor who was deceased (OR (*for every one-point increase in DASI score*) 0.97 (95% CI 0.96–0.99, *p* < 0.001).

**Table 2:** Post-operative outcomes after kidney transplantation and association with DASI score

	*N* (%)	OR (95% CI)*	*p*-value	aOR (95% CI)*^||^	*p*-value
Delayed graft function	109 (26.7)	0.98 (0.966–0.997)	0.019	0.99 (0.66–1.01)	0.359
Unplanned critical care admission	20 (4.9)	0.99 (0.96–1.02)	0.499	1.00 (0.97–1.04)	0.866
30-day hospital re-admission	96 (23.5)	0.99 (0.97–1.01)	0.240	1.01 (0.99–1.03)	0.457
30-day severe post-operative complication	70 (17.2)	0.99 (0.97–1.01)	0.289	1.01 (0.99–1.03)	0.489
30-day cardiovascular complication	8 (2)	0.99 (0.94–1.04)	0.605	0.99 (0.93–1.06)	0.850
		Co-efficient (95% CI)^±^		Adjusted co-efficient (95% CI)^±^^||^	
Length of stay, median (IQR), days	5 (4–7)	0.003 (0.001–0.004)	0.001	0.002 (0-0.003)	0.081

Key: IQR, interquartile range; OR, odds ratio; aOR, adjusted odds ratio; CI, confidence interval; DASI, Duke activity status index

*Results representing odds of experiencing complications for a one-unit increase in DASI scores

^±^ Results approximately representing percentage increase in logdays for a one-unit decrease in DASI scores

^||^ Adjusted for age, sex, dialysis vs. pre-emptive transplantation, presence of diabetes, deceased vs. living donor transplantation, cold ischaemic time

Data regarding all-cause mortality and death-censored graft loss are described in Table 3. Lower DASI scores were not associated with all-cause mortality or death-censored graft loss.

**Table 3:** Cox proportional hazards models assessing the association between DASI scores and all-cause mortality and death-censored graft loss following kidney transplantation

	*N* (%)	HR (95% CI)*	*p*-value	aHR (95% CI)* ^||^	*p*-value
All-cause mortality	24 (5.9%)	0.98 (0.95–1.01)	0.17	1.00 (0.97–1.03)	0.94
Death-censored graft loss	16 (3.9%)	0.97 (0.94–1.01)	0.10	0.97 (0.94–1.01)	0.17

Key: DASI, Duke Activity Status Index; HR, hazard ratio; aHR, adjusted hazard ratio; CI, confidence interval

*Results representing odds of experiencing complications for a one-unit increase in DASI scores

^||^ Adjusted for age, sex, dialysis vs. pre-emptive transplantation, presence of diabetes, deceased vs. living donor transplantation, cold ischaemic time

Subgroup analyses of live donor and deceased donor kidney transplant recipients are presented in Tables 4 and 5, respectively. After adjusting for confounders, the DASI score was not predictive of any adverse outcomes following kidney transplantation in either subgroup.

**Table 4:** Subgroup analysis of live donor kidney transplant recipients: association between DASI scores and adverse post-transplant outcomes

	*N* = 191	OR (95% CI)*	*p*-value	aOR (95% CI)*^||^	*p*-value
Delayed graft function (n, %)	10 (5.2)	0.98 (0.94–1.03)	0.47	0.83 (0.94–1.05)	0.83
Unplanned critical care admission (n, %)	9 (4.7)	1.00 (0.96–1.06)	0.84	1.03 (0.97–1.09)	0.37
30-day hospital re-admission (n, %)	38 (19.9)	0.99 (0.97–1.02)	0.44	1.00 (0.97–1.03)	0.91
30-day severe post-operative complication (n, %)	38 (19.9)	0.99 (0.96–1.01)	0.25	0.99 (0.96–1.02)	0.42
30-day cardiovascular complication (n, %)	3 (1.6)	0.97 (0.90—1.05)	0.44	0.96 (0.87–1.06)	0.37
		Co-efficient (95% CI)^±^		Adjusted co-efficient (95% CI)^±^^||^	
Length of stay, median (IQR), days	4 (4–7)	0.07 (-0.14-0.01)	0.07	0.06 (-0.13-0.02)	0.13
		HR (95% CI) *		aHR (95% CI) *^||^	
All-cause mortality	6 (3.1)	0.98 (0.93–1.04)	0.59	1.00 (0.94–1.06)	0.97
Death-censored graft loss	2 (1.0)	1.01 (0.90–1.12)	0.93	1.03 (0.90–1.17)	0.70

Key: IQR, interquartile range; OR, odds ratio; aOR, adjusted odds ratio; CI, confidence interval; DASI, Duke activity status index; HR, hazard ratio; aHR, adjusted hazard ratio

*Results representing odds of experiencing complications for a one-unit increase in DASI scores

^±^ Results approximately representing percentage increase in logdays for a one-unit decrease in DASI scores

^||^ Adjusted for age, sex, dialysis vs. pre-emptive transplantation, presence of diabetes, cold ischaemic time

**Table 5:** Subgroup analysis of deceased donor kidney transplant recipients: association between DASI scores and adverse post-transplant outcomes

	*N* = 217	OR (95% CI)*	*p*-value	aOR (95% CI)*^||^	*p*-value
Delayed graft function (n, %)	99 (45.6)	0.99 (0.98–1.01)	0.53	1.01 (0.98–1.03)	0.71
Unplanned critical care admission (n, %)	11 (5.1)	0.98 (0.93–1.02)	0.29	0.97 (0.92–1.02)	0.17
30-day hospital re-admission (n, %)	58 (26.7)	0.99 (0.97–1.02)	0.59	1.00 (0.97–1.02)	0.79
30-day severe post-operative complication (n, %)	32 (14.7)	0.99 (0.96–1.02)	0.48	0.99 (0.97–1.02)	0.70
30-day cardiovascular complication (n, %)	5 (2.3)	1.00 (0.94–1.07)	0.95	1.00 (0.93–1.07)	0.97
		Co-efficient (95% CI)^±^		Adjusted co-efficient (95% CI)^±^^||^	
Length of stay, median (IQR), days	5 (4–8)	-0.09-0.07	0.75	-0.05-0.12	0.41
		HR (95% CI) *		aHR (95% CI) *^||^	
All-cause mortality (n, %)	18 (8.3)	0.98 (0.95–1.02)	0.31	1.00 (0.96–1.04)	0.89
Death-censored graft loss (n, %)	14 (6.5)	0.97 (0.94–1.01)	0.17	0.97 (0.93–1.01)	0.14

Key: IQR, interquartile range; OR, odds ratio; aOR, adjusted odds ratio; CI, confidence interval; DASI, Duke activity status index; HR, hazard ratio; aHR, adjusted hazard ratio

*Results representing odds of experiencing complications for a one-unit increase in DASI scores

^±^ Results approximately representing percentage increase in logdays for a one-unit decrease in DASI scores

^||^ Adjusted for age, sex, dialysis vs. pre-emptive transplantation, presence of diabetes, cold ischaemic time

### DASI scores above 34

**Fig. 1:**
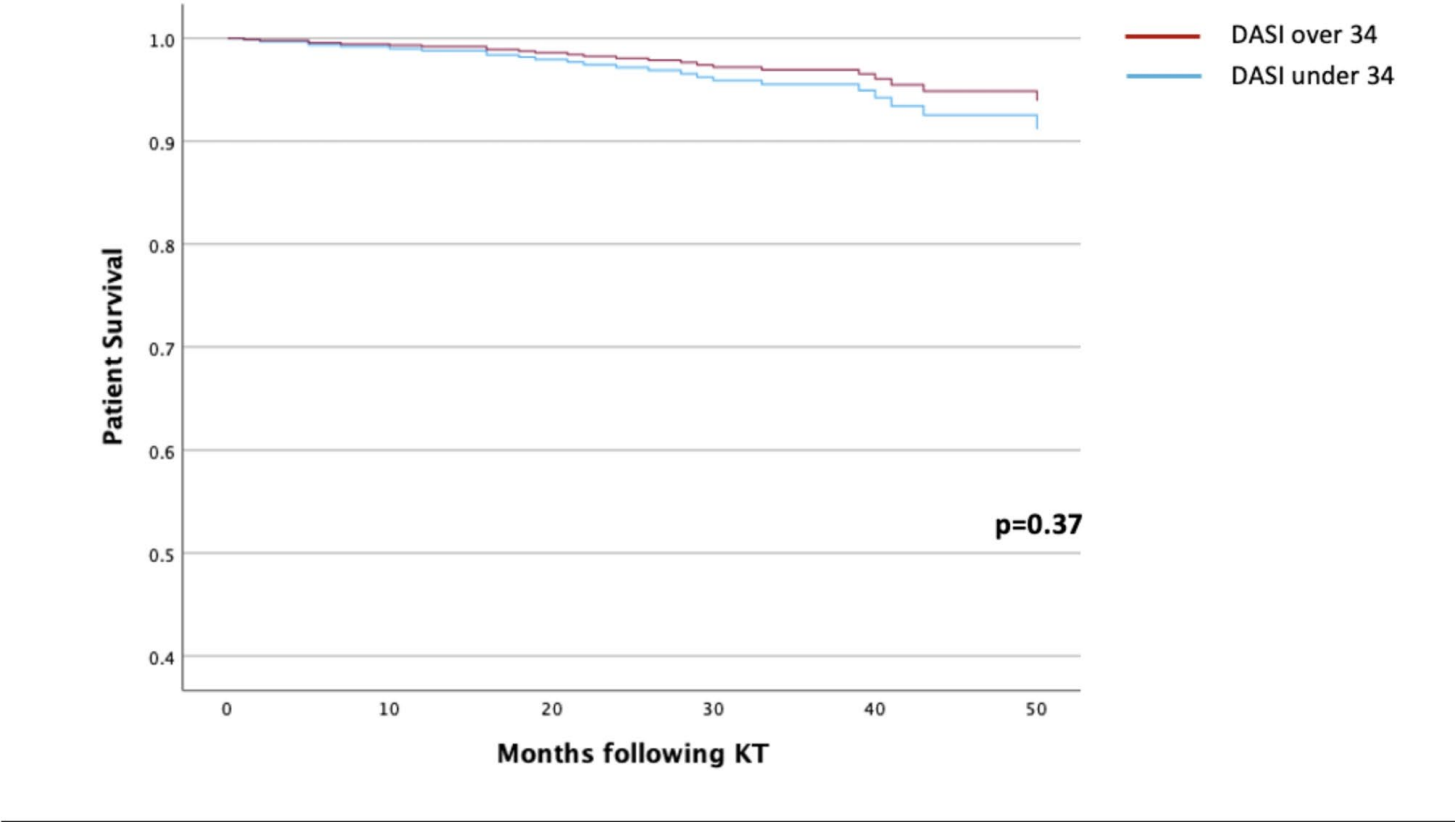
Adjusted patient survival curves following kidney transplantation stratified by DASI Score (≥ 34 vs. <34), based on cox proportional hazards regression. Key: DASI, Duke activity status index; KT, kidney transplantation. Adjusted for age, sex, dialysis vs. pre-emptive transplantation, presence of diabetes, deceased vs. living donor transplantation, cold ischaemic time

**Fig. 2:**
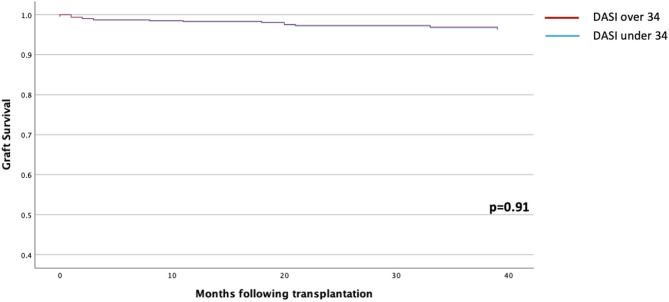
Adjusted death censored graft survival curves following kidney transplantation stratified by DASI Score (≥ 34 vs. <34), based on cox proportional hazards regression. Key: DASI, Duke activity status index. Adjusted for age, sex, dialysis vs. pre-emptive transplantation, presence of diabetes, deceased vs. living donor transplantation, cold ischaemic time

When DASI scores were dichotomized according to the cutoff determined by the METs study (> 34) [19], after adjusting for age, sex, dialysis vs. pre-emptive transplantation, presence of diabetes, deceased vs. living donor transplantation and cold ischemic time, DASI scores below 34 were not associated with any adverse outcomes (see Table 6; Figs. 1 and 2).

**Table 6:** Post-operative outcomes after kidney transplantation and association with DASI scores ≥ 34

	DASI > 34 *n* = 281	DASI < 34 *N* = 127	aOR(95% CI)*^||^	*p*-value
Delayed graft function	67 (23.8)	42 (33.1)	0.95 (0.52–1.72)	0.855
Unplanned critical care admission	11 (3.9)	9 (7.1)	0.54 (0.21–1.40)	0.205
30-day hospital re-admission	66 (23.5)	30 (23.8)	1.14 (0.68–1.90)	0.632
30-day severe postoperative complication	43 (15.3)	27 (21.3)	0.70 (0.40–1.22)	0.207
30-day cardiovascular complication	5 (1.8)	3 (2.4)	0.74 (0.17–3.31)	0.695
			Adjusted co-efficient (95% CI)^±^	
Length of stay, median (IQR)	5 (4–7)	5 (4–7)	-0.07 (-0.09-0.02)	0.172

Key: IQR, interquartile range; OR, odds ratio; aOR, adjusted odds ratio; CI, confidence interval; DASI, Duke activity status index

*Results representing odds of experiencing complications if DASI > 34

^±^ Results approximately representing percentage increase in logdays if DASI score > 34

^||^ Adjusted for age, sex, dialysis vs. pre-emptive transplantation, presence of diabetes, deceased vs. living donor transplantation, cold ischaemic time

## Discussion

In this single-centre retrospective cohort study DASI scores at the time of listing for transplantation did not independently predict short- or long-term adverse outcomes after transplant. DASI was predictive of delayed graft function and prolonged length of stay, however, after adjusting for confounders, this was no longer statistically significant. Of interest, patients with lower DASI scores were more likely to receive a transplant from a donor who was deceased. This is likely to be responsible for the association between lower DASI scores and delayed graft function and lower DASI scores and prolonged length of stay. To the best of our knowledge, this is the first study reporting on the ability of DASI to predict adverse outcomes for patients undergoing kidney transplantation. These findings suggest that the Duke Activity Status Index may have limited utility in assessing functional capacity in waitlisted kidney transplant candidates.

These findings are somewhat in contrast with what has been reported about DASI in the literature thus far. The METS study (*n* = 1401) did show that DASI was predictive of a statistically significant increased risk of death or myocardial infarction at 30 days (for every 3.5-point reduction in DASI aOR 0.91 95% CI 0.83–0.99, *p* = 0.03) [15]. However, in keeping with our study, it was not predictive of in-hospital moderate or severe complications or death at 1-year. Additionally, in the METS study, despite DASI being able to statistically predict those at increased risk of death or myocardial infarction at 30 days, when DASI was added to current risk prediction models, the area under the curve (AUC) was low at 0.67. Furthermore, it was unable to improve the ability to predict patients who would or would not go on to develop the outcome of interest [19], suggesting the impact of lower DASI scores on perioperative risk may be low. Hence, the clinical applicability of using DASI to inform decision-making on a patient’s suitability for surgery is questionable. Additionally, only 2% of the METS cohort had an eGFR under 30 ml/min/1.73m^2^ or were on dialysis. DASI has been assessed in cohorts of patients with chronic kidney disease [23–25], however, none of the patients in these studies required renal replacement therapy and the studies did not assess the ability of DASI to predict adverse outcomes after surgery. DASI, therefore, does not have construct validity in patients who are being considered for kidney transplant waitlisting. One of the potential reasons for this may relate to the subjective nature of the scoring system. A patient may over-report their ability to carry out the variety of activities assessed using the questionnaire if they deem this to have an impact on their chances of being waitlisted for a transplant. DASI may not be reflective of the true functional capacity of these patients.

Of interest, DASI has been used as part of the Fried Frailty Phenotype assessment tool, when used to assess frailty in solid organ transplant candidates [26]. The Fried Frailty Phenotype tool is based on five components: unintentional weight loss, weakness (measured by hand grip strength), exhaustion, low physical activity and slow walking speed. DASI has been used as a measure of low physical activity. Frailty, as measured by the Fried Frailty Phenotype, is predictive of adverse outcomes after kidney transplantation [27–31]. However, in studies that have reported the association of the individual components of the Fried Frailty Phenotype with adverse outcomes after transplantation [26], low physical activity was not predictive of adverse outcomes. The association of frailty and adverse outcomes after transplant was driven by exhaustion, slowed walking speed and reduced hand grip strength instead [32]. 

### Limitations

There are some limitations of this study. Firstly, this was a retrospective study design introducing the potential of selection bias in the cohort. However, with our centre having one of the highest rates per million population of kidney transplantation in the UK and Europe ([33, 34]), it is highly unlikely that the study population represents an inherently more robust cohort of patients. Additionally, the level of co-morbidity in our cohort was comparable, if not worse than that of the METS cohort, with 20% of recipients having a history of cardiovascular disease, 24% of recipients having a history of diabetes and 100% of patients having ESKD. Secondly, this study is from a single centre with a majority White population, limiting the applicability of our findings to other populations. However, the single centre nature of this study does avoid the potential bias that could emerge if multiple centres were using DASI scores in subtly different ways when deciding to waitlist patients for transplant. Thirdly, the event rates for 30-day cardiovascular complication, critical care admission, 1-year patient mortality and 1-year graft loss were low. The high rate of living donor transplantation, high rate of pre-emptive transplantation and use of an enhanced surgical recovery protocol [35] in our centre may account for this reduction in adverse clinical outcomes. Additionally, if the results from our analysis of DASI below and above 34 do indicate a trend to reduced survival, then it would take a study of nearly 4000 participants for this to be statistically significant. This would be a very ambitious recruitment target and likely require multiple centres in many countries to be achieved. The Canadian-Australasian Randomised Trial of Screening Kidney transplant candidates for coronary artery disease (CARSK) trial has encountered significant challenges in recruitment [36]. Despite commencement of recruitment in 2018, they are yet to reach their recruitment target of 3200 patients [37]. Finally, prospective serial measurements of DASI as opposed to a single baseline measurement may better correlate with outcomes, as a reduction or change in functional capacity may be more clinically relevant than a single snapshot measurement.

### Future work

We suggest that measures of functional capacity that are objective, reproducible, straightforward to administer and do not require significant financial or time resources be assessed in a prospective nature in patients being considered for waitlisting for kidney transplantation. One suggested measure would be the ability to climb two flights of stairs. In a prospective cohort study of 4559 patients, the inability to climb two flights of stairs was associated with adverse outcomes after major non-cardiac surgery and was able to improve the ability to predict whether a patient would or would not develop the outcome of interest [38]. In this study the ability to climb two flights of stairs relied on patient reporting however the subjective nature of this could be easily removed by assessing a candidate’s ability to do two flights of stairs in an outpatient setting, or with a video recording made at home.

### Conclusion

Reduced functional capacity increases the risk of adverse outcomes after kidney transplantation. However, in this retrospective cohort of recipients who underwent kidney transplantation, DASI scores at time of listing were not able to independently predict short- or long-term adverse outcomes. Further work is necessary to identify a measure of functional capacity in candidates being considered for waitlisting for transplantation that will be able to inform decision-making regarding transplantation and be easily implemented into clinical practice.

## Data Availability

Anonymized data is available upon request to the corresponding author. This is not publicly shared as clinical information is contained.

## Abbreviations

ADPKD: Autosomal dominant polycystic kidney disease
aHR: Adjusted hazard ratio
aOR: Adjusted odds ratio
BMI: Body mass index
CI: Confidence interval
CKD: Chronic kidney disease
CPET: Cardiopulmonary exercise testing
CV: Cardiovascular
DASI: Duke activity status index
DN: Diabetic nephropathy
eGFR: Estimated glomerular filtration rate
ESKD: End stage kidney disease
HLA: Human leucocyte antigen
HR: Hazard ratio
IQR: Interquartile range
METS: Measurement of Exercise Tolerance before Surgery
ml/min: Millilitre per minute
OR: Odds ratio
RRT: Renal replacement therapy
UK: United Kingdom
